# Supplementary material for: Rapid and high-resolution patterning of microstructure and composition in organic semiconductors using ‘molecular gates’
Source: Nat Commun. 2020 Jul 17;11:3610. doi: 10.1038/s41467-020-17361-8 (PMC7367850; doi:10.1038/s41467-020-17361-8)
Supplement: Supplementary file 1 — Supplementary Information [file 41467_2020_17361_MOESM1_ESM.pdf]

Supplementary Information

Rapid and high-resolution patterning of microstructure and composition in organic semiconductors using 'molecular gates'

Perevedentsev *et al*.

Supplementary Figures

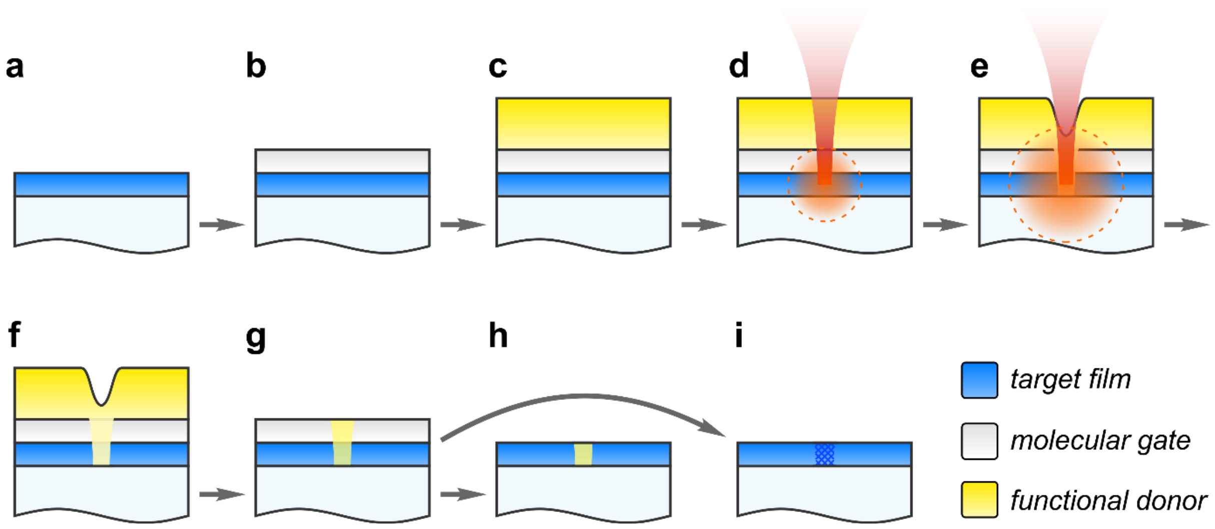

Supplementary Figure 1 | Schematic illustration of molecular-gate-based patterning. The sequential processing steps are shown for the exemplary case of laser-based patterning. (**a**) Deposition onto a substrate of the target organic semiconductor film. To enable light-to-heat conversion in transparent organics, the substrate may include an additional light-absorbing layer such as ITO. (**b**) Solution-based deposition of the molecular gate. If, for example, the target material is preferentially soluble in aromatic solvents (e.g. toluene or chlorobenzene), molecular gate material is then selected such that it can be deposited from solutions in aprotic or protic solvents. (**c**) Solution-based deposition of the donor layer comprising the functional small-molecular compound. If, for example, the molecular gate material is preferentially soluble in protic solvents (e.g. water or methanol), the donor layer is then deposited from solutions in orthogonal aprotic, aromatic or non-polar aliphatic solvents. (**d**) The laser is directed at the sample. In one configuration, the laser can be directed through the transparent substrate to minimise light-scattering within the crystalline donor layer that may lead to a deterioration of spatial resolution. In another configuration, the laser can be directed through the donor layer. The laser is focussed to a spot size that approximately corresponds to the desired spatial resolution. Light-to-heat conversion (depicted by the orange circle) occurs at the primary light-absorbing layer, e.g. the target semiconductor itself. (**e**) The generated heat propagates through the sample and raises the temperature at the donor layer such that the donor compound is melted or dissolved, therewith activating its diffusion into the target film via the molecular gate. (**f**) Laser excitation is then removed to arrest further diffusion of donor compound. (**g**) The residual donor layer is then removed by, e.g., exposure to the same solvents used for its deposition. (**h**) The molecular gate is then removed by, e.g., exposure to the same solvents used for its deposition. In case of patterning material composition, the donor compound remains within the patterned region of the target film. (**i**) Alternatively, in case of patterning microstructure (e.g. when donor comprises a small-molecular solvent), the target film may be further exposed to the same solvent used for donor layer deposition to finally remove the donor compound and retain the modified microstructure within the patterned region of the target film.

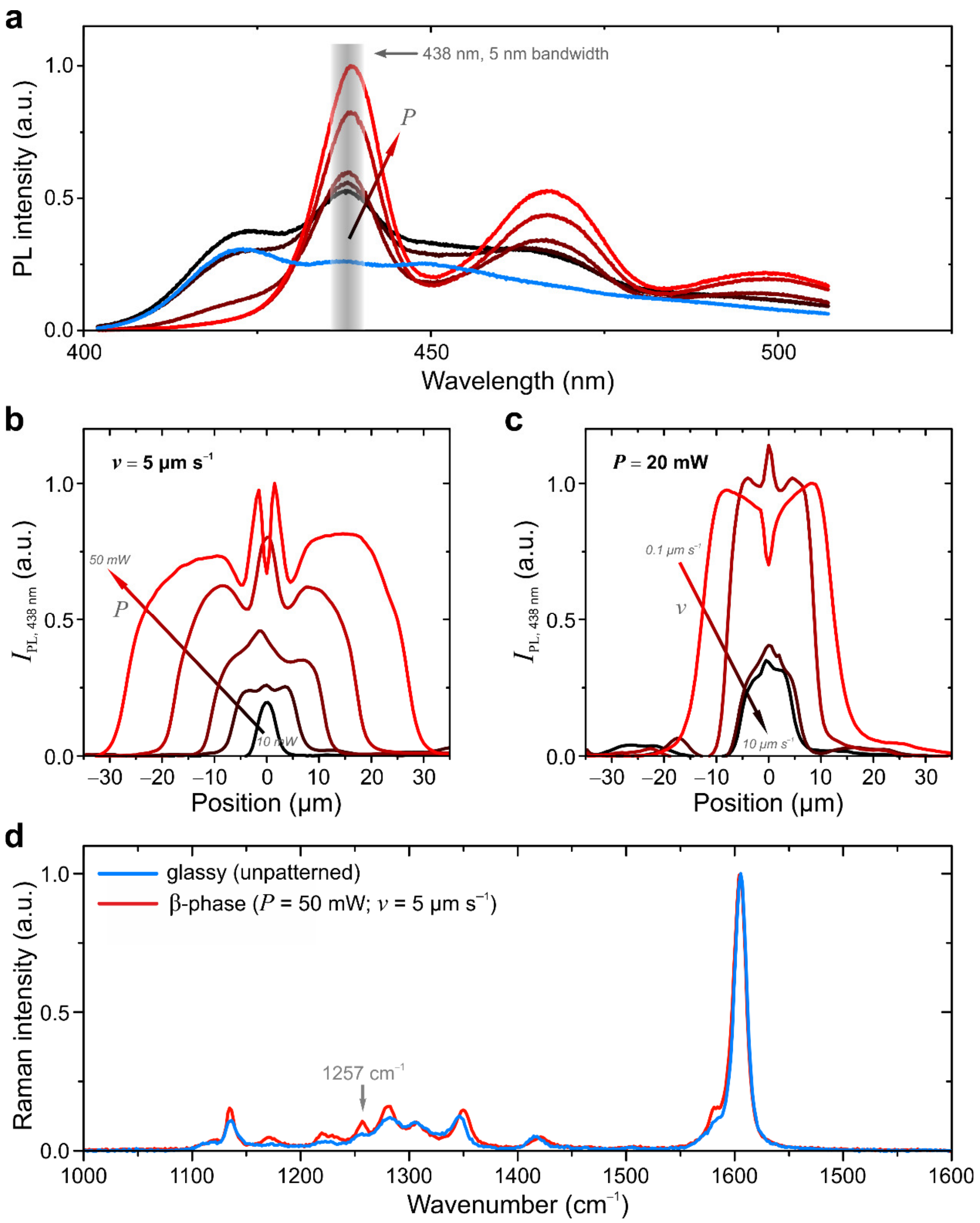

Supplementary Figure 2 | Analysis of β-phase line patterns. (**a**) PL spectra corresponding to the data in Fig. 2b in the main text, recorded at pattern locations featuring maximum induced β-phase fraction (black-to-red lines). Also shown is the PL spectrum recorded at a 'baseline', unpatterned area (blue line), corresponding to glassy PFO with a negligible β-phase fraction. (**b,c**) PL intensity, I_{PL}, profiles recorded for emission at 438 nm (5 nm bandwidth) across the β-phase lines patterned using (a) $v = 5$ μm s^{-1} and varied laser power and (b) $P = 20$ mW and varied writing speed. This data was used to estimate FWHM dimensions of β-phase line patterns (Fig. 2c,d in the main text). For clarity, the spectra are normalised by the maximum intensity of the highest-intensity spectrum ($P = 50$ mW). Grey shaded area indicates the spectral region for which the confocal PL images shown in Fig. 2b of the main text were recorded. (**d**) Raman spectra recorded for the pristine, glassy film area and the line pattern featuring the highest induced β-phase fraction (P = 50 mW; v = 5 μm s^{-1}).

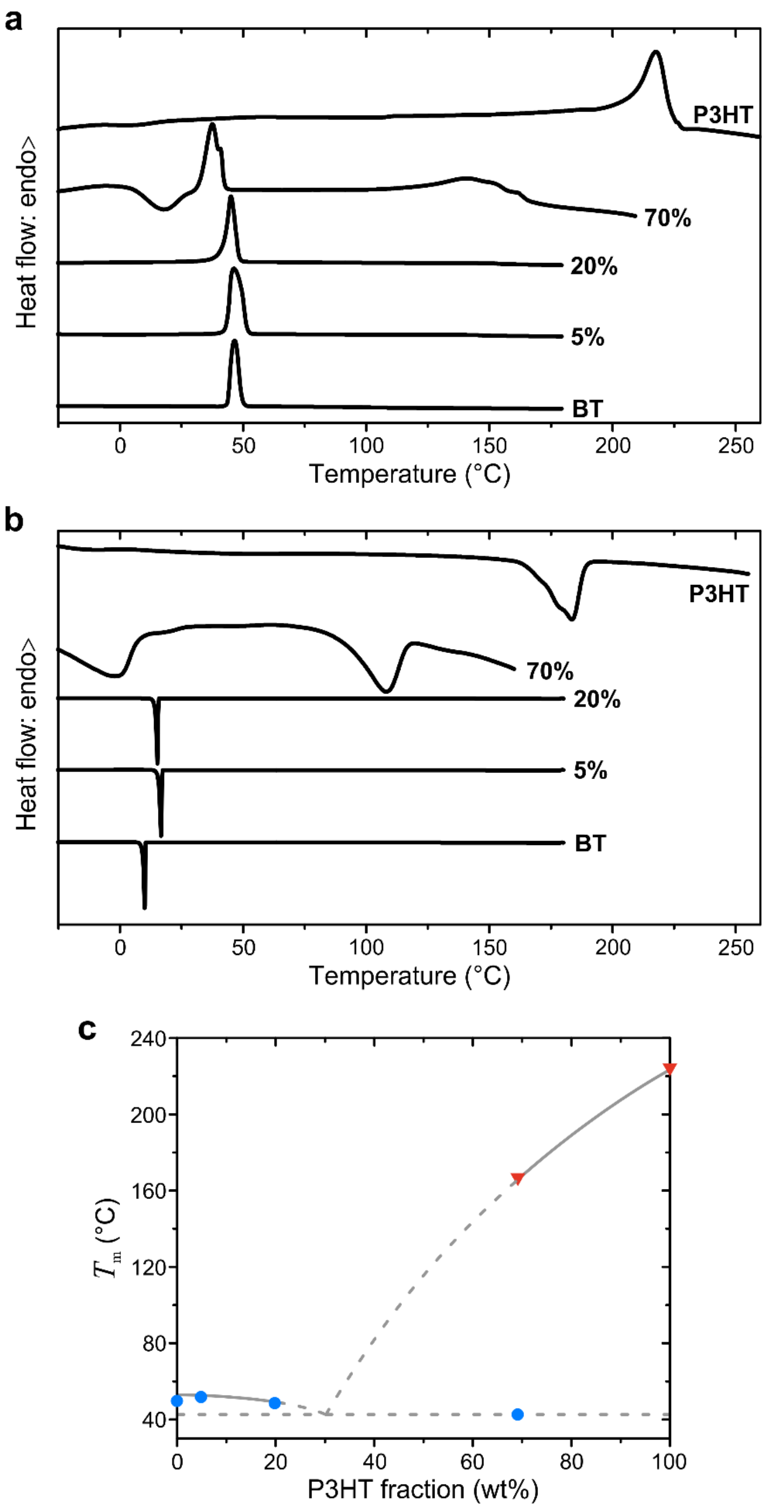

Supplementary Figure 3 | Phase behaviour of P3HT:BT blends. (**a**) Second-heating and (**b**) first-cooling thermograms recorded for P3HT:BT at various concentrations of P3HT (as indicated; accurate within ±1 wt%). (**c**) Temperature-composition 'phase' diagram for P3HT:BT, constructed using the respective endset dissolution/melting temperatures for low- (*blue circles*) and high-temperature (*red triangles*) transitions. Solid lines are guides to the eye for the observed transitions and dashed lines indicate their probable extensions. Note that, as shown in (b), for P3HT concentrations ≤20% both components crystallise *simultaneously* from the liquid phase as evident from the appearance of a single exotherm.

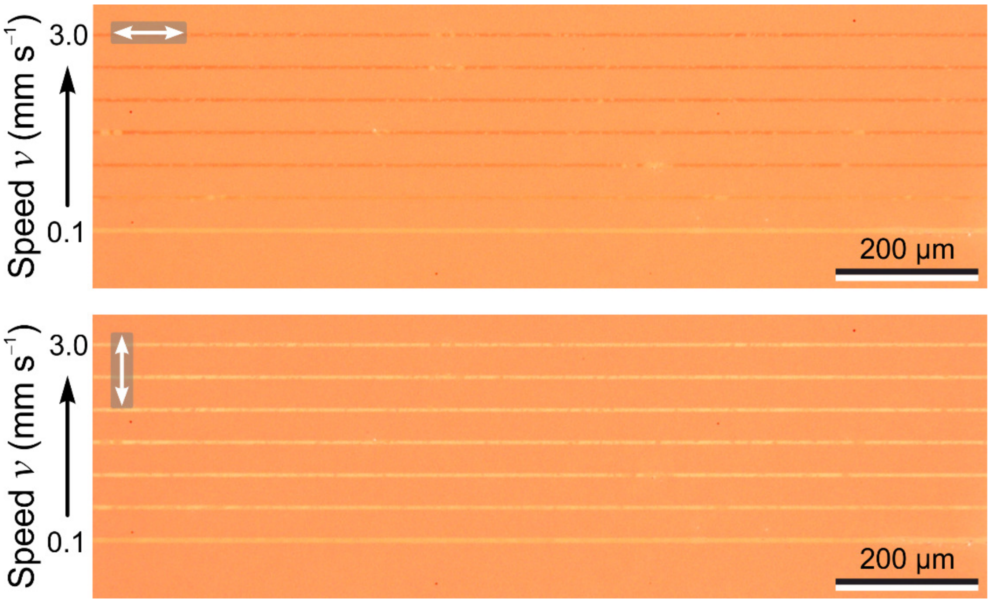

Supplementary Figure 4 | Large-area microscopy of chain orientation patterns. Polarised transmitted-light micrographs of a P3HT film laser-patterned at the indicated writing speeds v. Polarisation of incident light is indicated by the arrows.

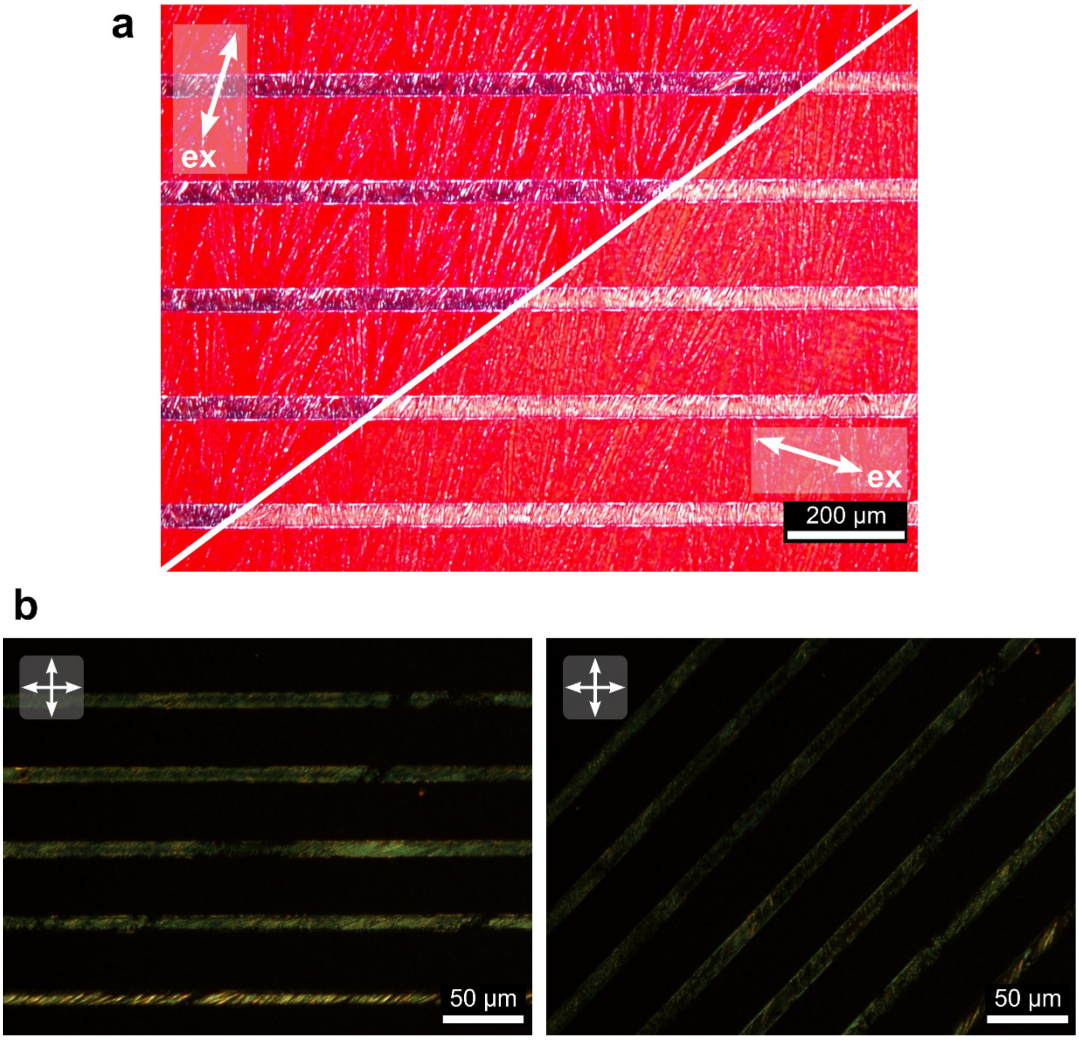

Supplementary Figure 5 | The necessity of molecular gate for laser-patterning chain orientation. (**a**) Polarised transmitted-light micrographs of a laser-patterned P3HT film for which the BT donor layer was spin-coated directly on top *without* the molecular gate. Polarisation of incident light is indicated by the arrows. (**b**) Cross-polarised micrographs (0° and 45° orientations) of a laser-patterned P3HT film using an *insufficiently thick* (<100 nm) molecular gate. As shown in (a), the absence of the molecular gate interlayer results in a change of film microstructure outside the patterned area, as well as the crystallisation of BT/P3HT being guided by the surface BT crystals rather than the laser scanning direction. The latter also applies in the case of an insufficiently thick molecular gate as shown in (b).

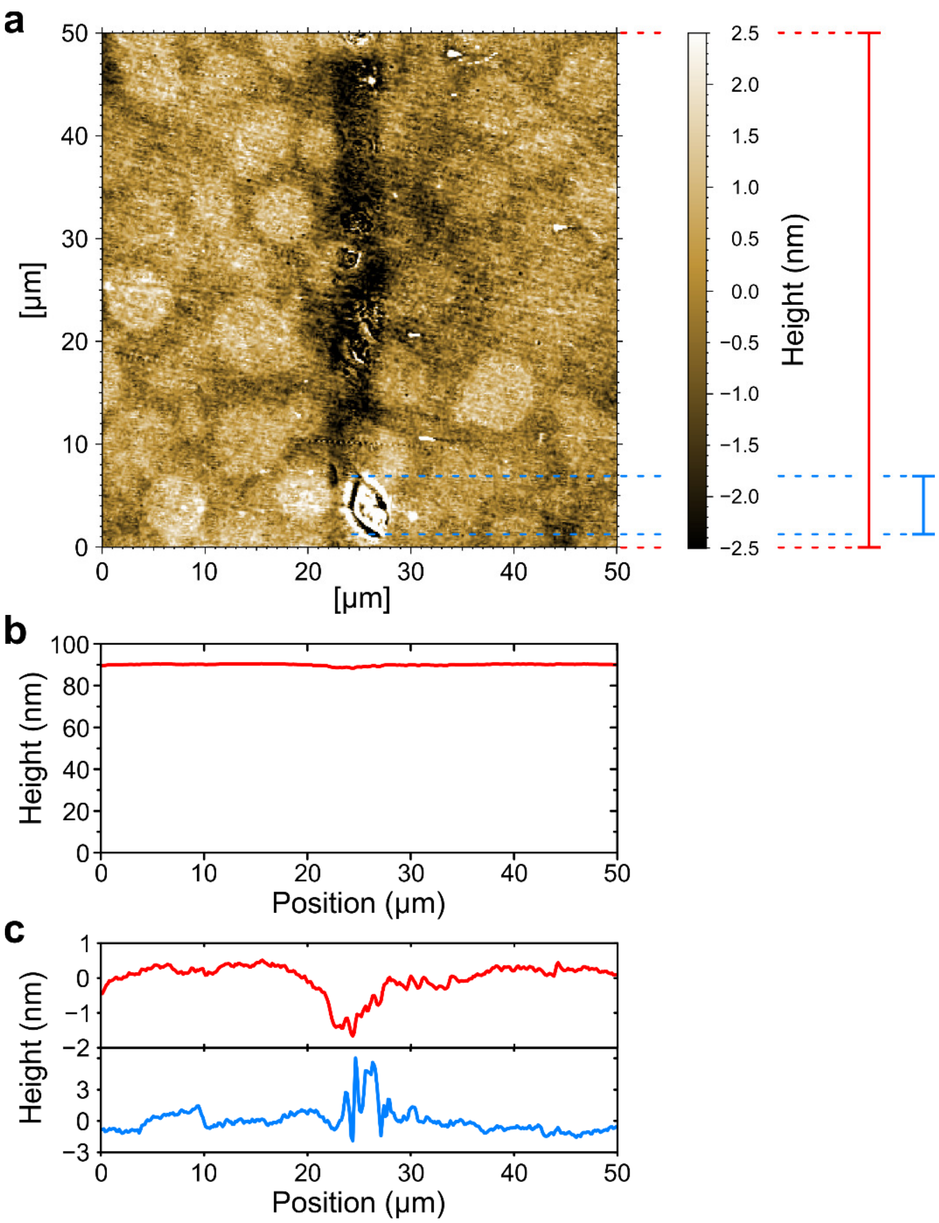

Supplementary Figure 6 | Molecular-gate-based patterning and film topography. (**a**) AFM image of the surface a 90-nm-thick in-plane isotropic P3HT film featuring a line-pattern of chain orientation extending vertically through the centre of the image (P = 55 mW, v = 2.5 mm s^{-1}; analysed in Fig. 3c–f in the main text). (**b**) Average topography profile across the pattern, shown here including the overall film thickness to highlight the retained planarity of patterned film. (**c**) Magnified topography profiles averaged across the whole AFM image (*red line*) and at the location of a surface defect (*blue line*), as indicated by the markers in (a). Note that the width of topographic dip in the upper panel of (c) matches the FWHM dimensions of the chain orientation line-pattern determined by polarised Raman mapping in Fig. 3e in the main text (~5 μm and 4.6 μm, respectively).

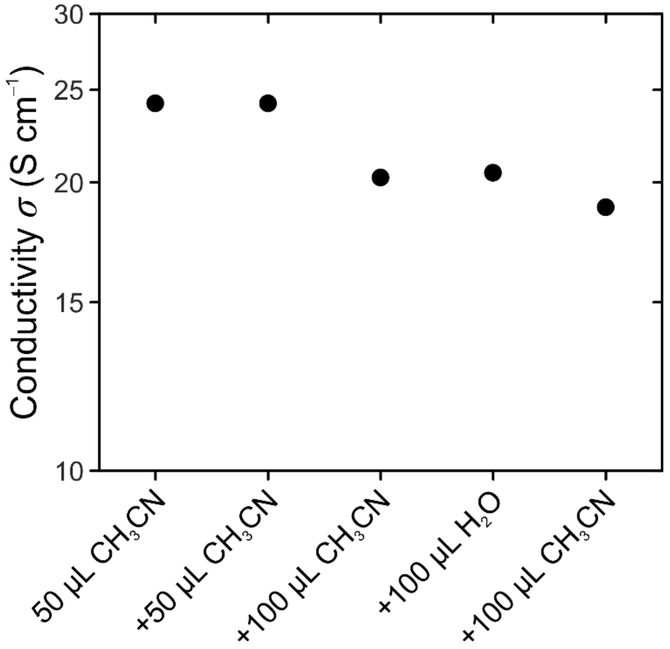

Supplementary Figure 7 | Spin-off stability test for a doped PBTTT film. Conductivity measured for a BCF-doped PBTTT film after sequential spin-off post-processing steps (deposited volumes indicated) in acetonitrile (CH_3CN) and water (H_2O). Exposure of an *as-doped* PBTTT trilayer sample to 50 µL CH_3CN—a good solvent of BCF—removes the residual dopant layer. Subsequent exposure of the *gate-protected* film to 150 µL CH_3CN marginally reduces the conductivity. Removal of the gate by spin-off using 100 µL H_2O does not change conductivity appreciably. Interestingly, further deposition of 100 µL CH_3CN onto an *unprotected* doped PBTTT film results in only a minor decrease of conductivity, indicating the high stability of BCF-doped PBTTT.

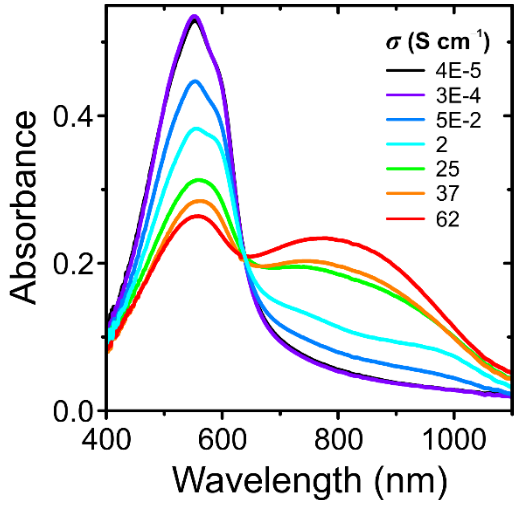

Supplementary Figure 8 | Absorption spectra of BCF-doped PBTTT films. Data is shown for samples doped by thermal annealing (see Fig. 4b,c in the main text). Electrical conductivities σ are indicated.

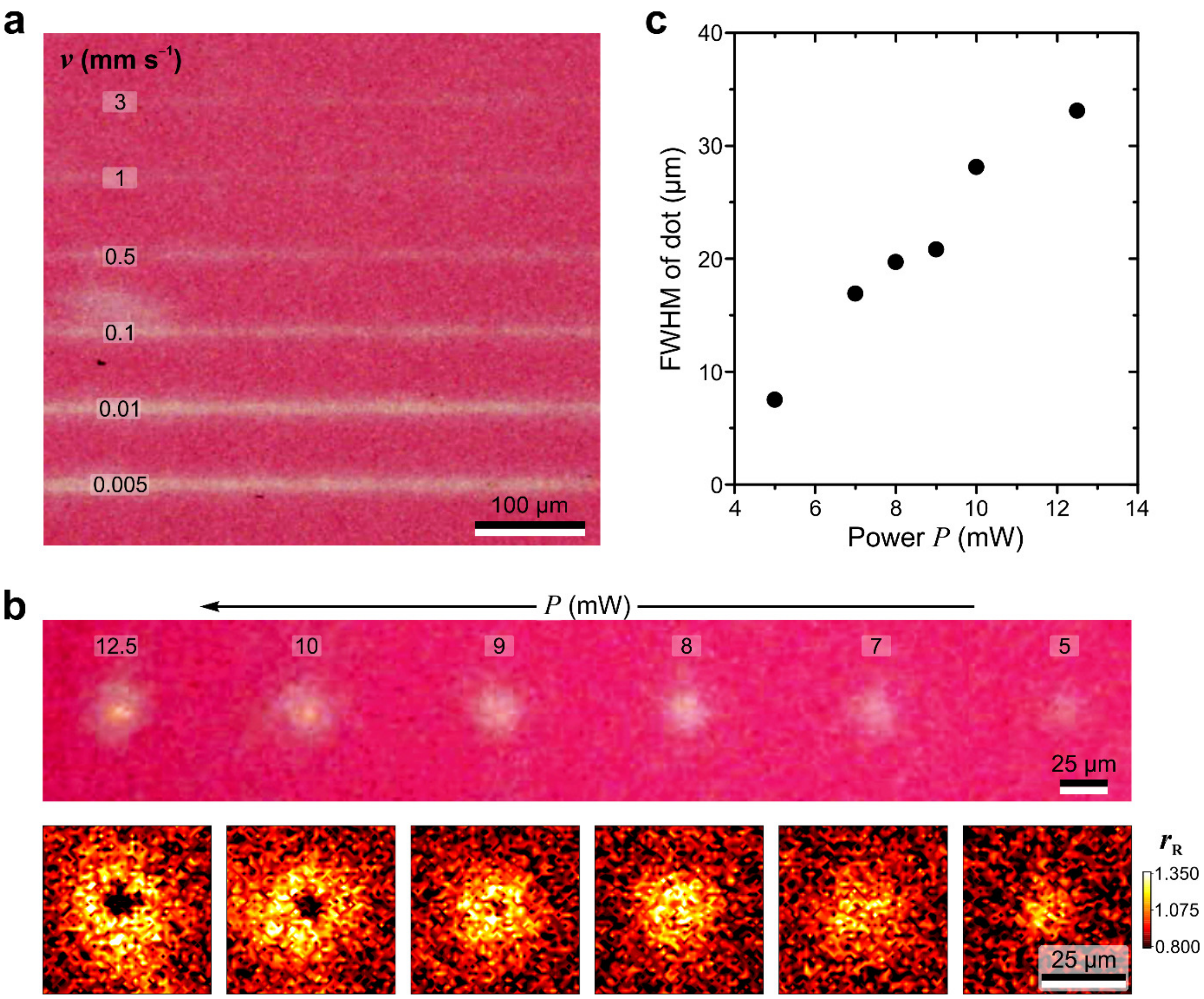

Supplementary Figure 9 │ Laser-patterning of BCF doping in PBTTT. (**a**) Exemplary transmitted-light micrographs of line-patterns of doped PBTTT written at P = 5 mW and the indicated speed v. (**b**) *Top panel*: transmitted-light micrographs of dot-patterns of doping in PBTTT (P as indicated, dwell time = 3 s); *bottom panel*: the corresponding Raman intensity ratio maps (r_R = ratio of maximum intensities of the ~1393 and ~1417 cm^{-1} peaks of PBTTT). Note the overheating-induced de-doping at the centres of dots patterned at P = 9–12.5 mW. (**c**) FWHM diameters of the dots in (b) as a function of P.

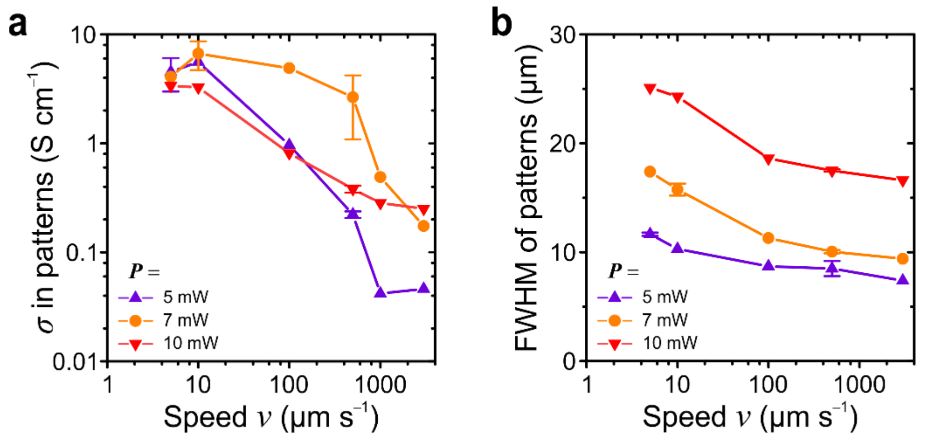

Supplementary Figure 10 │ Variation of pattern characteristics with laser writing parameters. Data is shown for BCF-doped PBTTT films. (**a**) Maximum electrical conductivities σ (reproduced from Fig. 4f in the main text) and (**b**) FWHM dimensions for lines written using scanning laser excitation at 532 nm for the indicated laser power P and writing speed v. In both cases, pattern characteristics were obtained by spectroscopic Raman mapping (see main text for details).

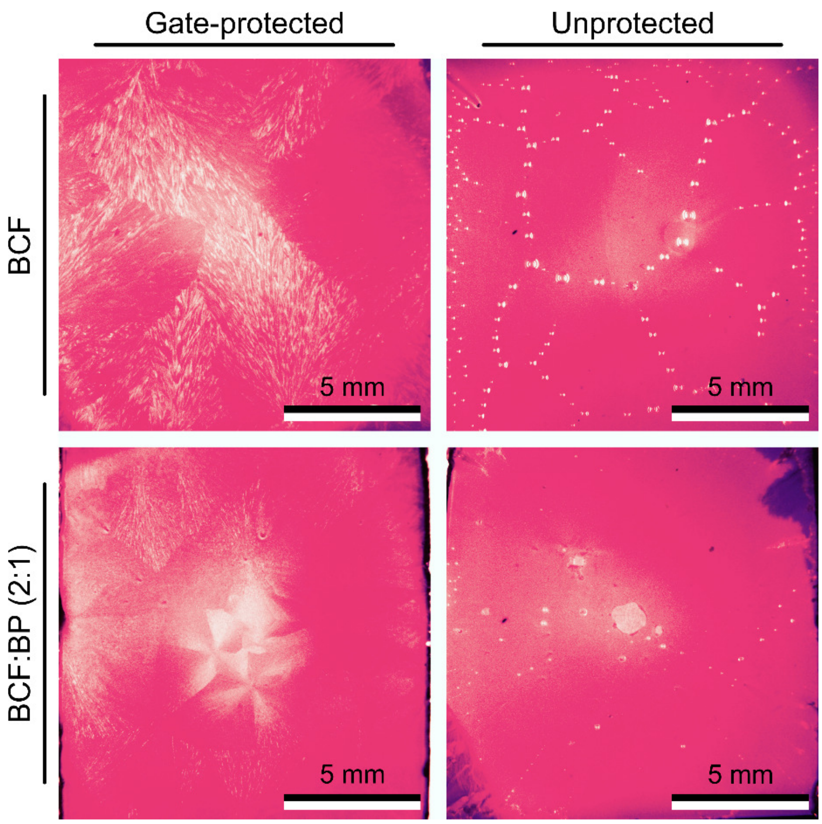

Supplementary Figure 11 │ Compatibilising function of the molecular gate. Transmitted-light micrographs of PBTTT films with and without a spin-coated pNaSS interlayer, onto which solutions of BCF and BCF:BP were spin-coated (as indicated). The absence of the hydrophilic pNaSS interlayer prevents formation of macroscopically coherent donor films on PBTTT (i.e. surface-crystals thereof), instead yielding isolated dewetted regions.

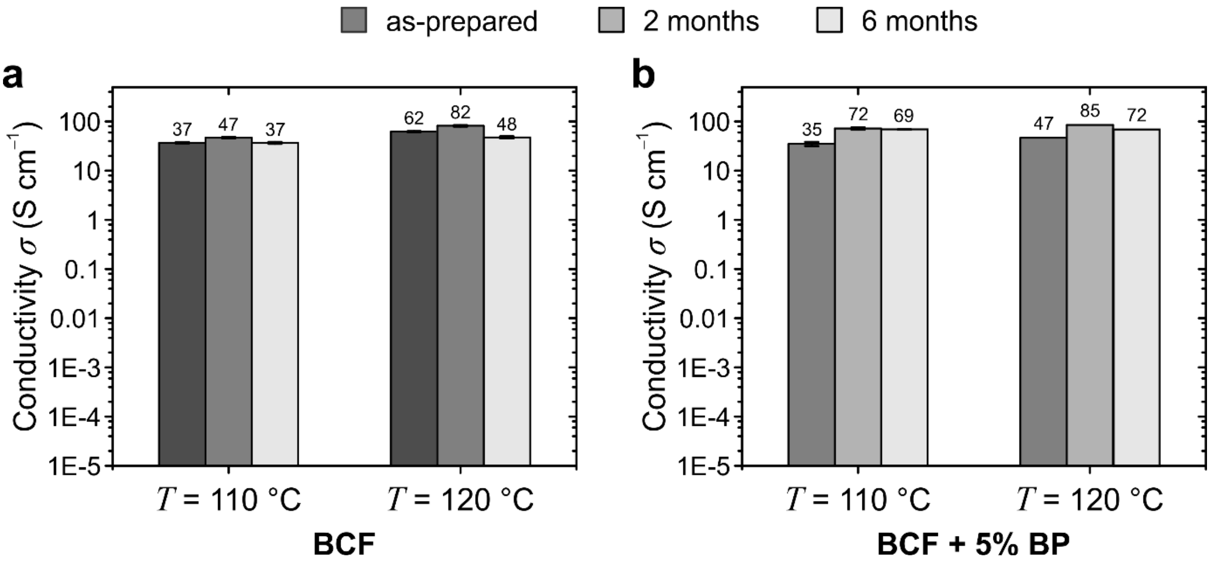

Supplementary Figure 12 │ Long-term stability of PBTTT films doped by BCF via the molecular gate. Conductivity σ of PBTTT films doped by solution-deposited dopant layers comprising (**a**) BCF and (**b**) BCF + 5 wt% biphenyl (BP) added as a co-solvent, followed by annealing for 1 min at the indicated temperatures T (as in Fig. 4b in the main text). Data is shown for as-obtained films, and the same films following aging in ambient atmosphere for 2 and 6 months. In each case, the corresponding error bars (from measurements on different sample areas) and average conductivities are indicated.

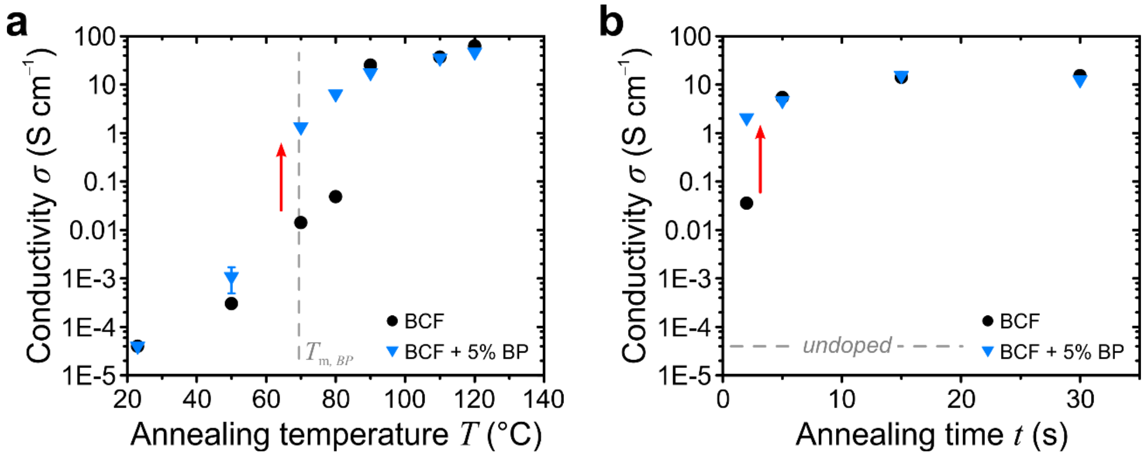

Supplementary Figure 13 │ Optimising thermally-induced BCF doping of PBTTT using a co-solvent. Conductivity σ of PBTTT films doped by (**a**) annealing for 1 min at temperature T (reproduced from Fig. 4b in the main text for ease of reference) and (**b**) annealing at 100 °C for time t. Solution-deposited dopant layers comprised BCF and BCF + 5 wt% biphenyl (BP) added as a co-solvent. Dashed line in (a) indicates the melting temperature of BP. Dashed line in (b) indicates the conductivity of pristine, undoped PBTTT. Red arrows highlight the changes in the evolution of σ with annealing temperature or time induced by the presence of a small amount of BP. Note that, as expected, the solvating effect of BP is manifested only at annealing temperatures above its melting point ($T_m \approx 69$ °C), as seen in (a).

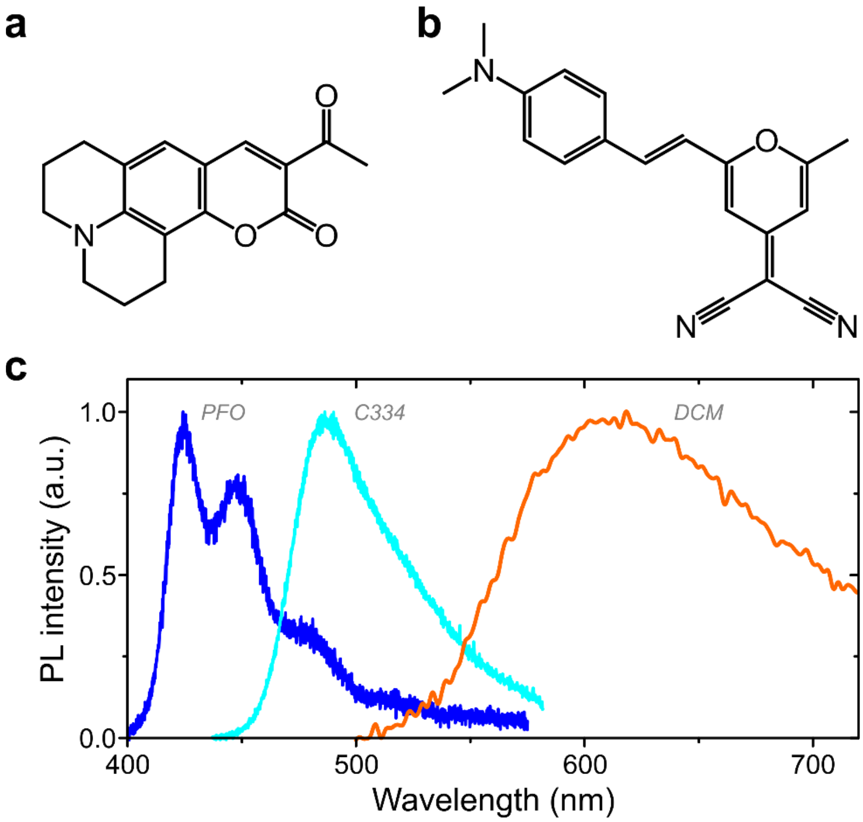

Supplementary Figure 14 | Chemical structures and reference PL spectra of PFO and individual dyes. Chemical structures of (**a**) C334 and (**b**) DCM dyes (chemical structure of PFO is shown in Fig. 1c in the main text). (**c**) PL spectra recorded with excitation at 365 nm for glassy PFO as well as C334 and DCM dyes (dispersed in a PMMA matrix).

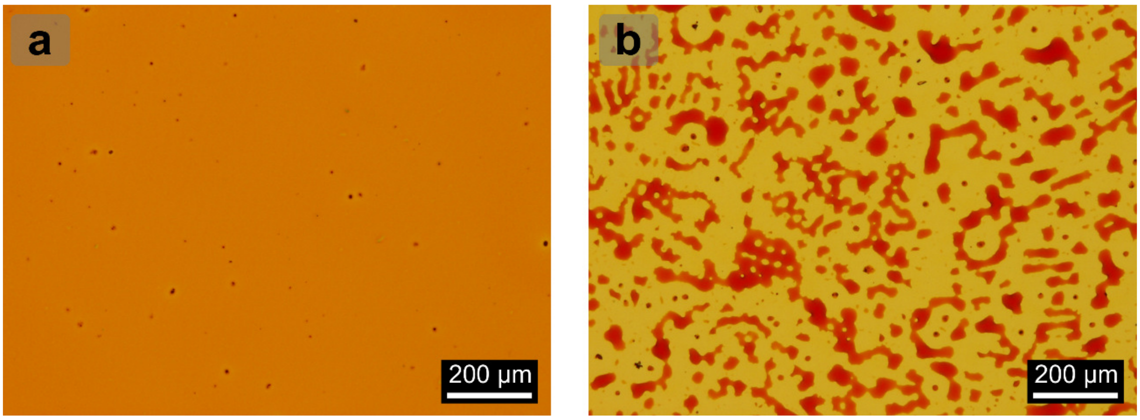

Supplementary Figure 15 | Effect of PMMA matrix for solvent-vapour-induced dye diffusion. Transmitted-light micrographs of the PFO/pNaSS/dye-blend trilayer after exposure to solvent vapour. Micrographs are shown for samples in which the dye-blend donor layer (**a**) contained PMMA and (**b**) did *not* contain PMMA. Clear dewet ting of the dye-blend donor layer is seen in the absence of the PMMA matrix, thereby preventing reproducible patterning.

Supplementary Tables

	**Laser power P =**				
	**5 mW**	**7 mW**	**8 mW**	**9 mW**	**10 mW**
**v (µm s^{-1})**	FWHM dimensions (µm)				
3000	7.4	9.4	12.3	15.1	16.6
5	11.7	17.4	20.2	22.0	25.1
×600	×1.6	×1.9	×1.6	×1.5	×1.5

	**Writing speed v =**				
	**3000 µm s^{-1}**	**500 µm s^{-1}**	**100 µm s^{-1}**	**10 µm s^{-1}**	**5 µm s^{-1}**
**P (mW)**	FWHM dimensions (µm)				
5	7.4	8.5	8.7	10.3	11.7
10	16.6	17.5	18.6	24.3	25.1
×2	×2.2	×2.1	×2.1	×2.4	×2.2

Supplementary Table 1 | Effect of laser power P and writing speed v on pattern dimensions. Data is shown for BCF-doped PBTTT films (see Fig. 4f in the main text) for the maximal span of writing speeds (3000 to 5 µm s^{-1}; *upper table*) and maximal span of laser powers (5 to 10 mW; *lower table*). FWHM dimensions of doping patterns were obtained by spectroscopic Raman mapping (see main text for details). In each case, the factor difference is given in red.

Supplementary Notes

Supplementary Note 1 │ Details of solvents and additional materials used.

- Toluene (>99.8%; Labkem)
- Dichloromethane (99.9%, stabilised with amylene; Labkem)
- Diethyl ether (>99.5%, stabilised with BHT; Labkem)
- Acetone (>99.5%; Sigma-Aldrich)
- Ethyl acetate (99.8%, anhydrous; Sigma-Aldrich)
- Methanol (>99.6%; Sigma-Aldrich)
- Acetonitrile (>99.8%, anhydrous; Sigma-Aldrich)
- Chlorobenzene (>99.5%, Honeywell)
- Triton X-100 surfactant (laboratory grade; Sigma-Aldrich)
- Water (deionised, Milli-Q)
